# Supplementary material for: A new testudinoid turtle from the middle to late Eocene of Vietnam
Source: PeerJ. 2019 Feb 18;7:e6280. doi: 10.7717/peerj.6280 (PMC6383559; doi:10.7717/peerj.6280)
Supplement: Supplemental Information 1 [file peerj-07-6280-s001.docx]

**A NEW TESTUDINOID TURTLE FROM THE MIDDLE TO LATE EOCENE OF VIETNAM AND ITS IMPLICATION FOR GEOEMYDID SYSTEMATICS**

Rafaella C. Garbin, Madelaine Böhme, Walter G. Joyce

**SUPPLEMENTARY MATERIAL**

**Appendix S1.** List of morphological characters with comments on their use and definitions

In this study we updated the character matrix of Garbin, Ascarrunz & Joyce (2018) by adding 16 new characters. Below we give the definition of these new characters and their references. For a list of the remaining characters refer to Appendix S2 of Garbin, Ascarrunz & Joyce (2018).

**Character Nº2**. Carapace, median keel, presence; *0 = present, perceptible ridge present or inflection in the intervertebral sulci; 1 = absent* (modified from Garbin, Ascarrunz & Joyce, 2018; characters 1 and 2)

**Comments:** To better document variation found to the development of keels seen in geoemydid turtles (Claude & Tong, 2004), we split the two characters of Garbin, Ascarrunz & Joyce (2018) that pertain to carapacial keels into six characters. In particular, carapacial keel characters are separated into ones that address the median and lateral keels separately, followed by aspects regarding number, extent, height continuity and position.

Carapacial keels fade into a smooth carapace during the ontogeny of many geoemydids, but traces nevertheless are always retained in the form of median or lateral ridges on the carapace and/or inflection along the posterior margins of each vertebral and pleural scute, which trace the former presence of keels. We consider both characteristics (i.e., the ridge itself and a trace of the ridge) to be evidences for the presence of carapacial keels.

**Character Nº4**. Carapace, median keel, height continuity; *0 = same height (continuous keel); 1 = variable height (discontinuous keel)* (modified from Garbin, Ascarrunz & Joyce, 2018; characters 1 and 2)

**Comments:** The median carapacial keel of testudinoids can either have a continuous height throughout its extent, or it can vary its height in some particular regions due to the presence of a projection of the keel (e.g., at the posterior region of the third vertebral scute in *Pangshura* species), or the keel can completely be interrupted at a particular bone and continue later along the same axis. We consider the two latter cases to be a situation where the keel is varying in height (state 1), independently if it is because of an interruption of the keel or a projection.

**Character Nº5**. Carapace, lateral keels, presence; *0 = present, perceptible ridge present or inflection in the intervertebral sulci; 1 = absent* (modified from Garbin, Ascarrunz & Joyce, 2018; characters 1 and 2)

**Comments:** *See character 2 above.*

**Character Nº6**. Carapace, lateral keels, position and extent on carapace; *0 = anterior half (pleural scutes I–II); 1 = anterior and posterior halves (pleural scutes I–IV); 2 = posterior half (pleural scutes III–IV); 3 = central (pleural scutes II–III)* (modified from Garbin, Ascarrunz & Joyce, 2018; characters 1 and 2)

**Comments:** We created this character based on variation observed in our sample of extant geoemydid specimens. In some specimens that have lateral keels, these keels can have a longer or shorter extent and vary in their position. This character is scored as inapplicable if lateral keels are not observed.

**Character Nº7**. Carapace, lateral keels, position relative to neural series; *0 = about half way between neural series and peripherals; 1 = closer to the neural series than peripheral series* (modified from Claude and Tong, 2004, 32, 2bis)

**Comments:** Claude & Tong (2004) noticed that *Palaeoemys testudiniformes*, *Geoclemys hamiltonii* and *Malayemys* spp. have lateral carapacial keels that are closer to the vertebral scutes than those of other geoemydids. We here investigate the presence of this character in other species by fully describing it relative to the carapacial bones.

**Character Nº27**. Carapace, cervical scute, notch on posterior margin; *0 = absent; 1 = present* (new character)

**Character Nº28**. Carapace, cervical scute, median keel; *0 = absent; 1 = present* (new character)

**Comments for characters 27 and 28:** Variation is apparent to the morphology of the cervical: in some specimens there is a notch (i.e., invagination) at the posterior margin of this scute whereas others have a median keel that is continuous with the main median keel of the carapace. Although these two characters may seem correlated (as the presence of an invagination on the sulcus could indicate the trace of a carapacial ridge), no correlation was observed (see Appendix S4 for the scorings).

**Character Nº29**. Carapace, nuchal, ventral view, curved protrusion posterior to first marginal; *0 = absent; 1 = present* (new character)

**Comment:** In visceral view, specimens referable to *Bridgeremys pusilla* exhibit a thick lip on the nuchal bone made by the first marginal scutes. Just posterior to this lip, there is a step, followed by a deep concavity on the nuchal. It is reminiscent of the epiplastral lip, which is common in many testudinoids, but on the nuchal and with a deep median invagination (Figure S1.1).


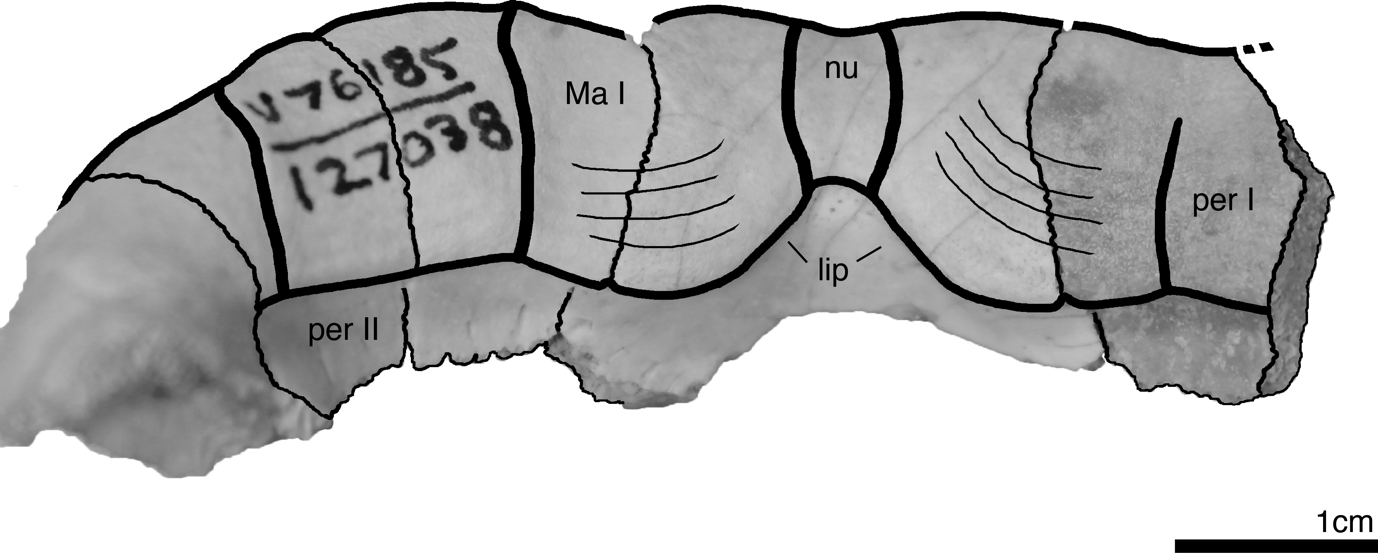


**Figure S1.1:** Visceral view of the anterior plastron margin of Bridgeremys pusilla UCMP 127038 showing the rounded nuchal lip at the center of the image. Legend: per – peripheral bones; nu – nuchal; lip – nuchal lip; Ma – marginal scutes.

**Character Nº65**. Bridge, fifth marginal scute, mid-lateral extension over hyoplastron; *0 = absent; 1 = present* (modified from Garbin, Ascarrunz & Joyce, 2018, character 57)

**Character Nº67**. Bridge, sixth marginal scute, mid-lateral extension over hyoplastron; *0 = absent; 1 = present* (modified from Garbin, Ascarrunz & Joyce, 2018, character 58)

**Comments for characters 65 and 67:** We developed these two characters to extend the information we had before on the overlapping of the fifth and sixth marginal scutes onto the plastron (i.e., characters 57 and 58; Garbin, Ascarrunz & Joyce, 2018). Besides reaching/contacting the hyo and hyoplastra, in some specimens of geoemydids the fifth and sixth marginal scutes clearly overlap these mid-plastron bones through a mid-lateral extension. Both characters where scored as present only in *Batagur dhongoka* and *Pangshura tecta*, while the extension of the sixth marginal scute was more frequent, and scored as present in many geoemydid species.

**Character Nº69**. Bridge, hyo-hypoplastron suture, contact with peripheral; *0 = peripheral V; 1 = between peripheral V and VI; 2 = peripheral VI* (New character)

**Comments:** The hyo-hypoplastron suture can contact peripheral VI (as in *Pangshura tecta* and *Rhinoclemmys annulata*), peripheral V (as in *Orlita borneensis*, *Siebenrockiella crassicollis* and *Malayemys* spp.), and both peripherals in *Geoemyda spengleri*. This character could not be observed in the fossils analyzed in this study.

**Character Nº73**. Plastron, gularohumeral sulcus, notch at anterior margin; *0 = absent; 1 = present* (New character)

**Comments:** We developed this character to document variation observed in some specimens of geoemydid (extant and fossil) that clearly have a notch/inflection at the anterior plastral margin, at the most lateral portion of the gularohumeral sulcus. We consider the notch as present if the angle created by this notch is smaller or equal to 120^o^.

**Character Nº74**. Plastron, gular scute, relation between length and width; *0 = longer than wide; 1 = as long as wide; 2 = wider than long; 3 = twicer wider than long* (modified from Claude & Tong, 2004, 39)

**Comments:** Claude & Tong (2004) mentioned that most Cretaceous and Paleocene testudinoids have a short gular scute (i.e., wider than long), and that more recent testudinoids developed a derived condition of longer gular scutes that overlap the entoplastron. This is a rather important character for geoemydids of the “*Palaeoemys group*”, which are characterized by gular scute that are much wider than long (Hervet, 2004). In this study, the presence of gular scutes that are wider than long is retrieved as a synapomorphy of crown Geoemydidae, which, however, is reversed in some groups.

**Character Nº75**. Epiplastron, visceral view, gular lip, extent; *0 = closer to anterior margin (shorter than the mid-length of epiplastra); 1 = around the mid-length of epiplastra (around 50%); 2 = closer to entoplastron margin (longer than the mid-length of epiplastra)* (modified from Claude & Tong, 2004, 40)

**Character Nº77**. Epiplastron, visceral view, step posterior to gular lip; *0 = absent; 1 = present* (modified from Claude & Tong, 2004, 40)

**Character Nº78**. Epiplastron, visceral view, gular lip, medial contact; *0 = present, gular lips meet at midline; 1 = absent, gular lips do not meet* (new character)

**Comments for characters 75, 77 and 78:** The presence of a gular lip on the visceral side of the epiplastron is a common characteristic in testudinoid turtles, especially in crown Testudinidae where many species have a thick and overhanging lip followed by a posterior step/excavation (Claude & Tong, 2004; Joyce & Bell, 2004). Although not as thick, in geoemydids the presence of an epiplastral lip is also common and we developed three characters here to better document the variation observed. These characters are scored as inapplicable if an epiplastral lip is absent.

When present, independent of thickness, the gular lip of the epiplastron can have a variable length: short, as in *Rhinoclemmys* spp*.* (closer to the anterior plastron margin); long, as in many testudinids, *Echmatemys septaria*, and *Bridgeremys pusilla* (closer to the entoplastron); or intermediate, as some *Cuora* spp. (about as long as the median of the epiplastra). We note that the gular lips of some specimens are locally near the lateral margins of the epiplastra and do not have a midline contact (Figure S1.2).

Many testudinids (i.e., *Geochelone*, *Chelonoidis*, *Stigmochelys*; Claude & Tong, 2004) have an “excavation” posterior to the gular lip. Here we consider this excavation to be a step (or concavity), due to the thickness change from the gular lip to the continuation of the epiplastra (Figure S1.3). The presence of this step is then correlated to the presence of a thick gular lip.

**Character Nº93**. Xiphiplastron, visceral view, lateral lip; *0 = absent; 1 = present* (new character)

**Comments:** Like the gular lip on the epiplastra, a lip can be formed by the femoral and anal scutes on the visceral side of the xiphiplastra. This lip is scored as present when its thickness is considerably different from the rest of the xiphiplastra. This character was scored as present in *Rhinoclemmys pulcherrima* and many fossils including *Echmatemys* spp., *Br. pusilla*, *G. pingi*, *Mauremys thanhinensis* and one specimen of *Ba. trani.* We unfortunately could not score this character for most extant species as we do not have access to pictures of the visceral surface of this bone.


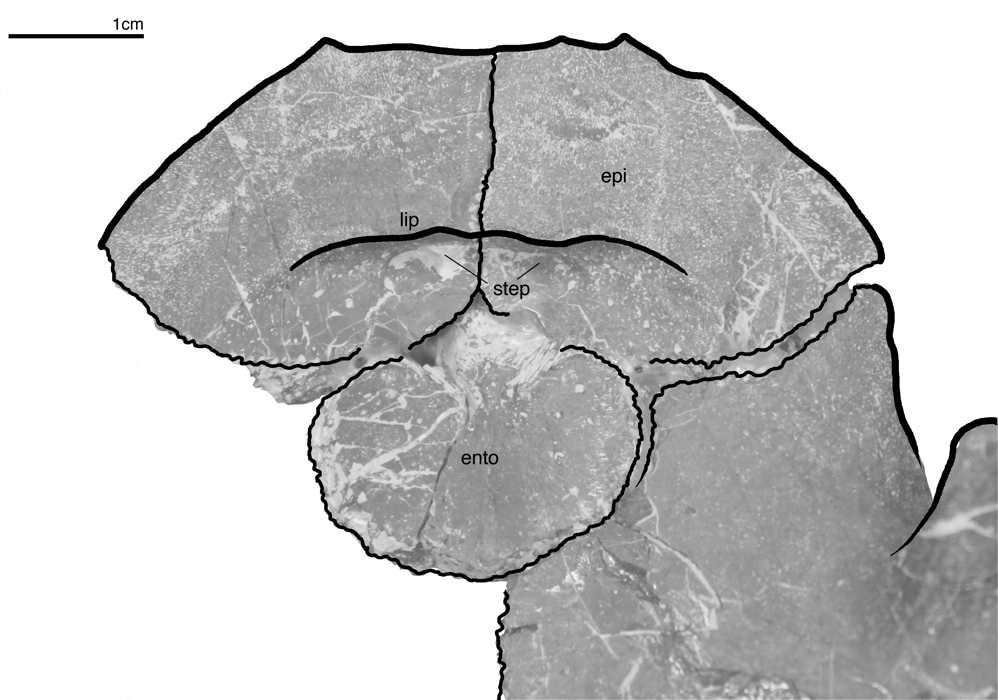


**Figure S1.2:** Visceral view of the anterior plastron lobe of Bridgeremys pusilla UCMP 127034 showing the gular folding lip on the epiplastra and its step. Legend: epi – epiplastral bones; ento – entoplastron; lip – gular folding lip.


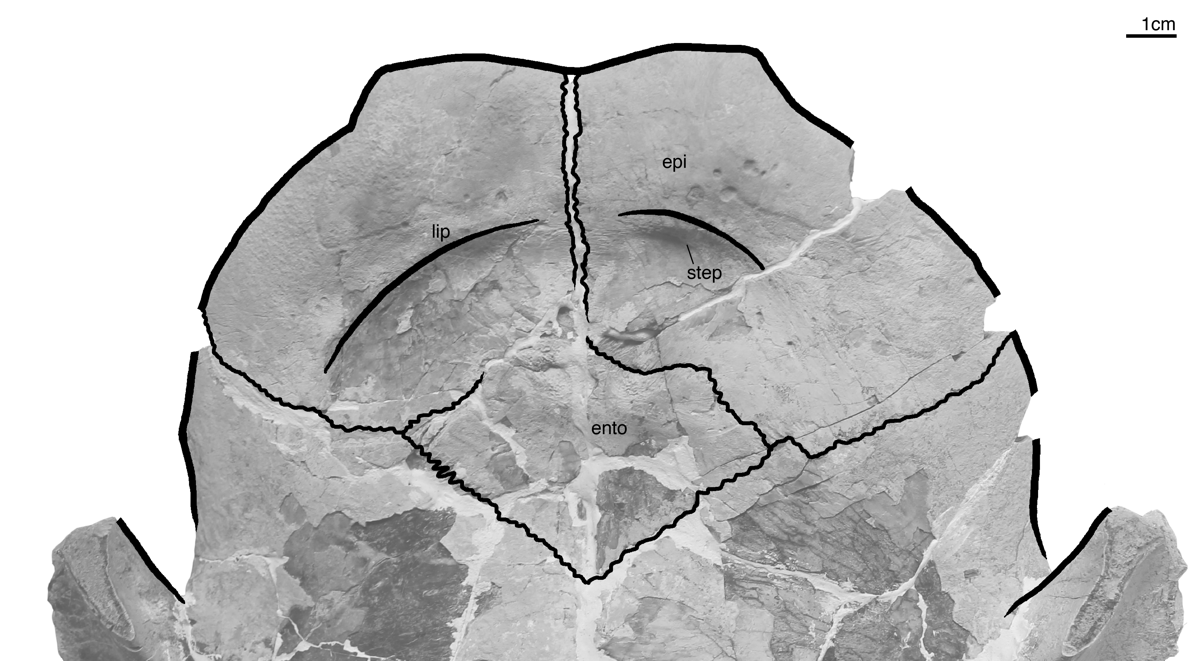


**Figure S1.3:** Visceral view of the anterior plastron lobe of Echmatemys wyomingensis UCMP PR720 showing the gular folding lip of the epiplastra that do not contact at midline. Legend: epi – epiplastral bones; ento – entoplastron; lip – gular folding lip.
