# Supplementary material for: A new testudinoid turtle from the middle to late Eocene of Vietnam
Source: PeerJ. 2019 Feb 18;7:e6280. doi: 10.7717/peerj.6280 (PMC6383559; doi:10.7717/peerj.6280)
Supplement: Supplemental Information 2 [file peerj-07-6280-s002.docx]

**A NEW TESTUDINOID TURTLE FROM THE MIDDLE TO LATE EOCENE OF VIETNAM AND ITS IMPLICATION FOR GEOEMYDID SYSTEMATICS**

Rafaella C. Garbin, Madelaine Böhme, Walter G. Joyce, others XXXX

**SUPPLEMENTARY MATERIAL**

**Appendix S2.** Specimens examined.

The specimens analyzed in this study are deposited in the following institutions and collections: American Museum of Natural History (AMNH) – herpetology collection; Eberhard Karls University of Tübingen (EKUT) – vertebrate paleontology collection; Field Museum of Natural History (FMNH) – herpetology and vertebrate paleontology collections; Muséum d’Histoire Naturelle de la Ville de Genève (MHNG) – herpetology collection; Museum für Tierkunde Dresden (MTD) – herpetology collection; Peter C. H. Pritchard collection, Chelonian Research Institute (PCHP); National Museum of Natural History (USNM) – herpetology collection; Naturhistorisches Museum Basel (NMB) – herpetology collection; Naturhistorisches Museum Wien (NMW) – herpetology collection; University of California Museum of Paleontology (UCMP) – vertebrate paleontology collection.

A complete list of all extant material analyzed for this study is given in Appendix S1 of Garbin, Ascarrunz & Joyce (2018). Here we list only new material added to their matrix, mostly extinct material. All other fossils species not included in this list were analyzed through description in the literature.

**Geoemydidae**

*Banhxeochelys trani* – Testu03, Testu06, Testu07, Testu08, Testu10, Testu12, Testu23, Testu24, Testu25, Testu26, Testu32, Testu34, Testu35, Testu39, Testu40, Testu41, Testu42, Testu43, Testu44, Testu45, Testu46, Testu51, Testu56, Testu60, Testu1001, Testu1002, Testu1004, Testu1005, Testu1007, Testu1011, TestuY1.

*Bridgeremys pusilla* – UCMP 127026, 127030, 127034, 127038, 127051.

*Echmatemys septaria* – FMNH PR606, PR709, PR816, PR1561.

*Echmatemys wyomingensis* – FMNH PR280, PR607, PR720, UR455, UR456.
