# Supplementary material for: A new testudinoid turtle from the middle to late Eocene of Vietnam
Source: PeerJ. 2019 Feb 18;7:e6280. doi: 10.7717/peerj.6280 (PMC6383559; doi:10.7717/peerj.6280)
Supplement: Supplemental Information 5 [file peerj-07-6280-s005.docx]

**A NEW TESTUDINOID TURTLE FROM THE MIDDLE TO LATE EOCENE OF VIETNAM AND ITS IMPLICATION FOR GEOEMYDID SYSTEMATICS**

Rafaella C. Garbin, Madelaine Böhme, Walter G. Joyce

**SUPPLEMENTARY MATERIAL**

**Appendix S5.** Consensus tree including fossil geoemydids

The consensus of the geoemydid phylogeny including each fossil species at a time is given below from figures S1 to S12.


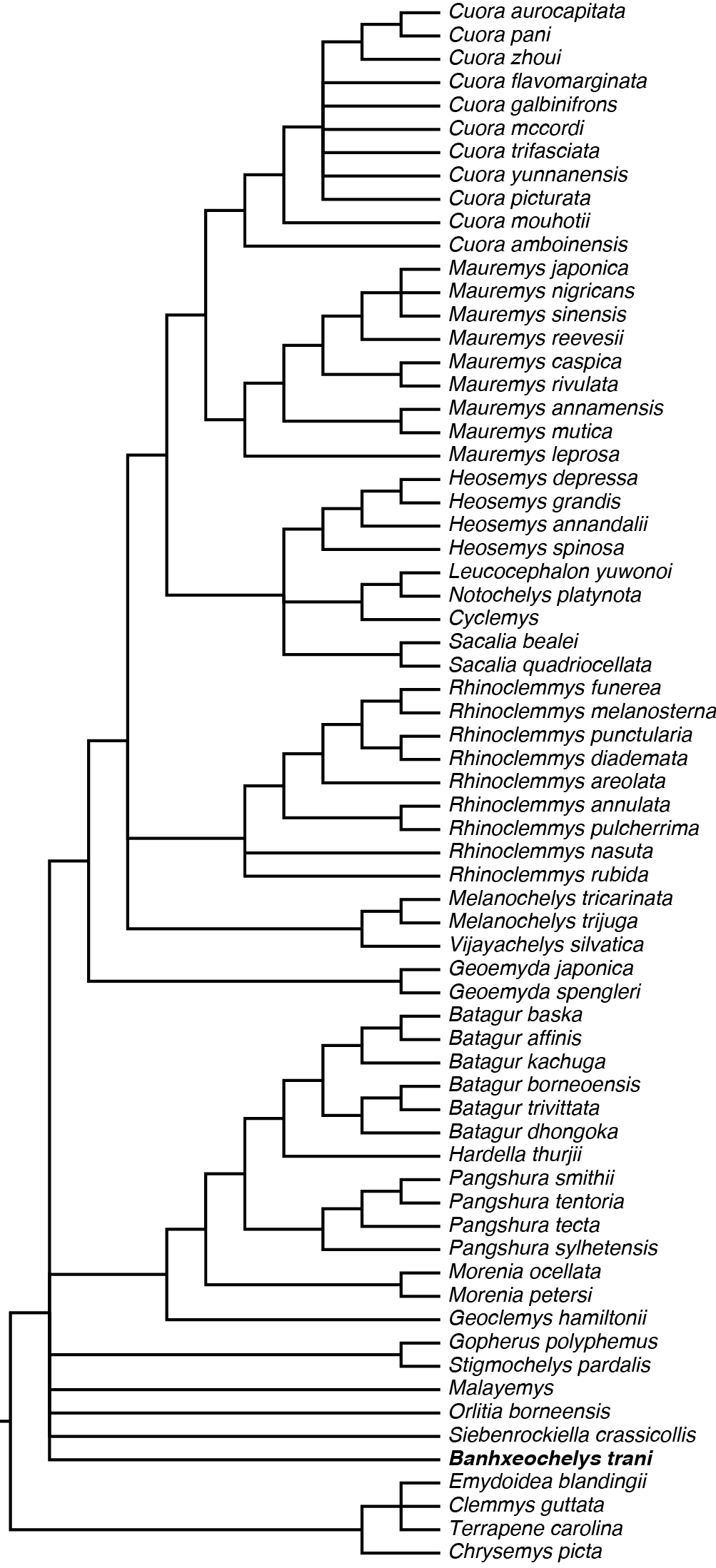


Figure S5.1: Strict consensus of total-evidence analysis with inclusion of the fossil testudinoid *Banhxeochelys trani* gen. et sp. nov.

Figure S5.2: Strict consensus of total-evidence analysis with inclusion of the putative fossil geoemydid *Bridgeremys pusilla*.


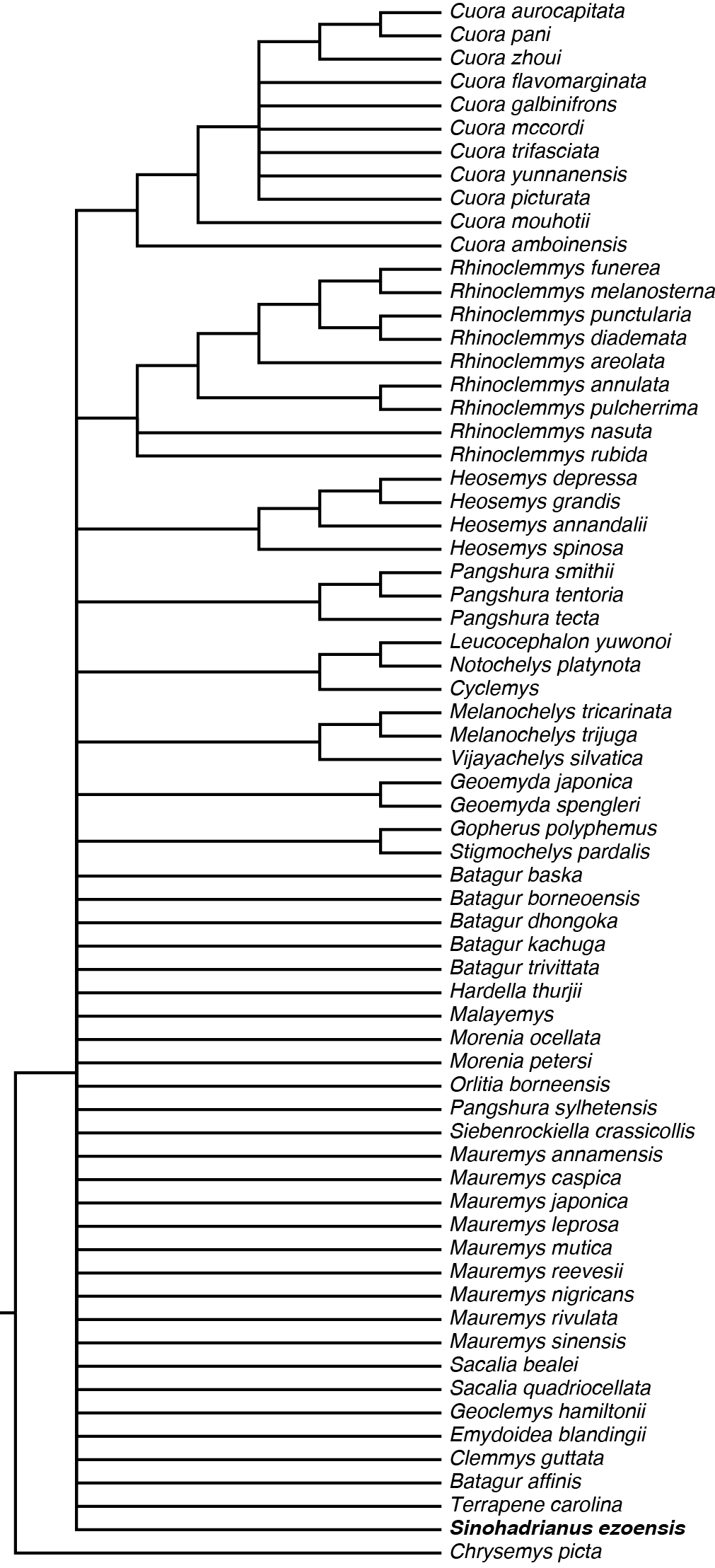


Figure S5.3: Strict consensus of total-evidence analysis with inclusion of the putative fossil testudinoid *Sinohadrianus ezoensis*.

Figure S5.4: Strict consensus of total-evidence analysis with inclusion of the putative fossil geoemydid *Isometremys lacuna.*


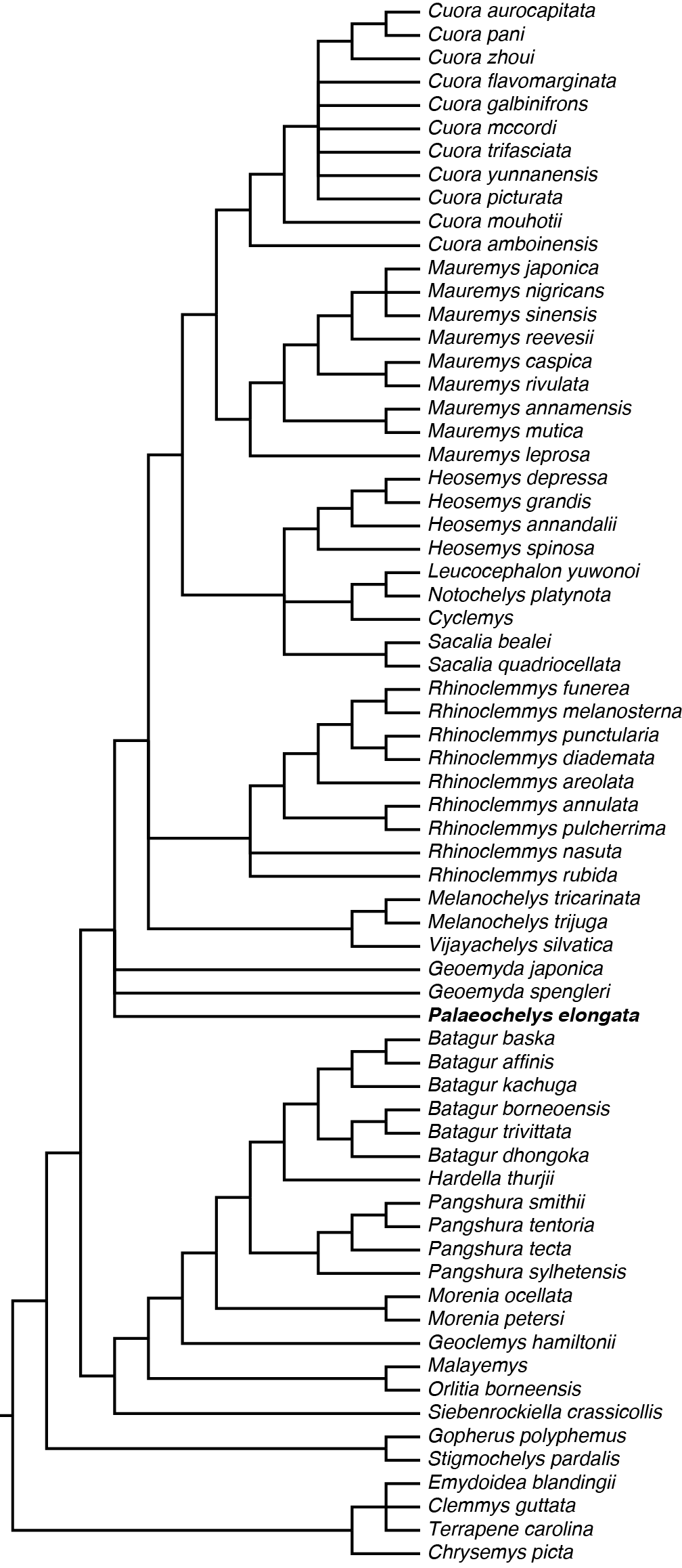


Figure S5.5: Strict consensus of total-evidence analysis with inclusion of the putative fossil geoemydid *Palaeochelys elongata*.

Figure S5.6: Strict consensus of total-evidence analysis with inclusion of the putative fossil geoemydid *Guangdongemys pingi*.


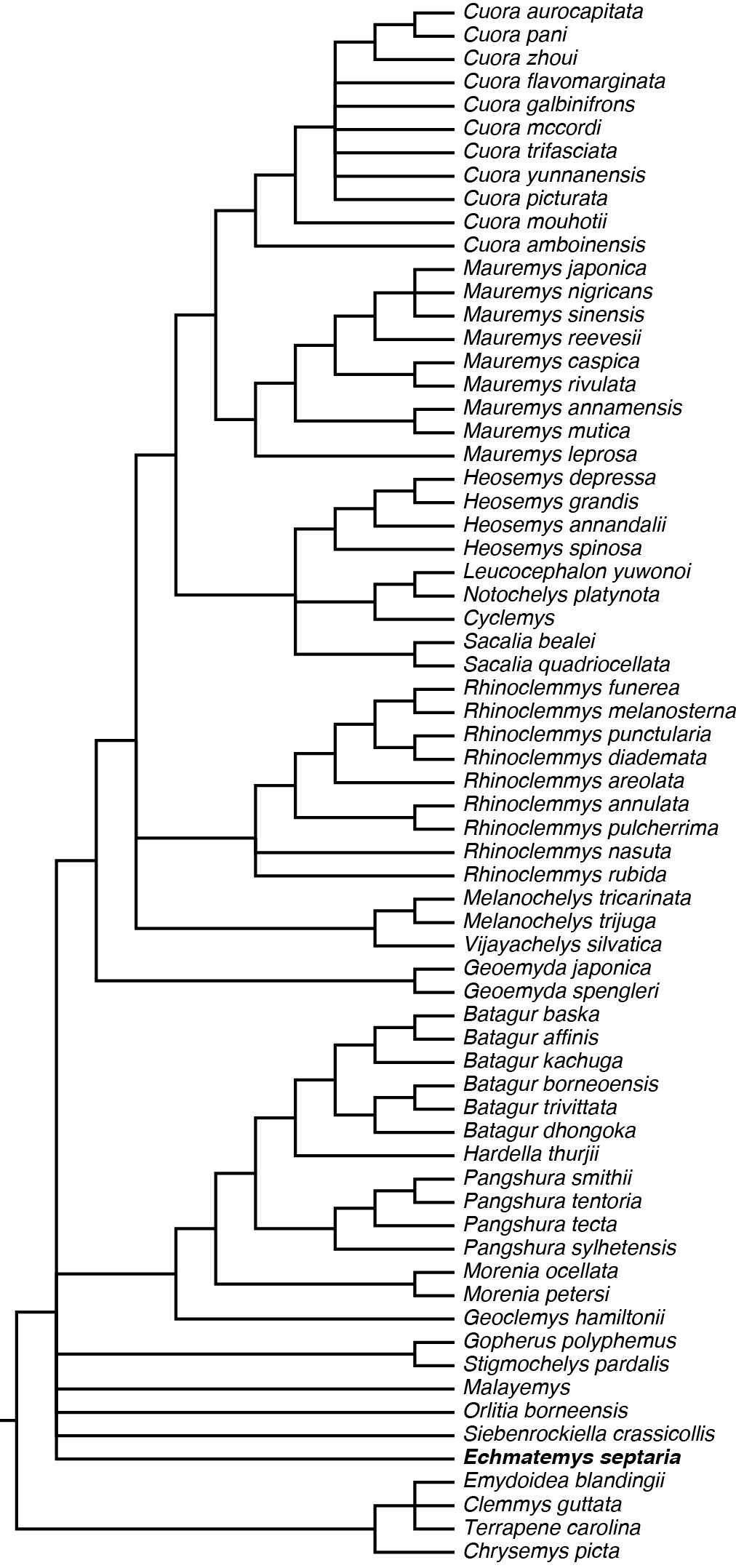


Figure S5.7: Strict consensus of total-evidence analysis with inclusion of the putative fossil geoemydid *Echmatemys septaria*.

.
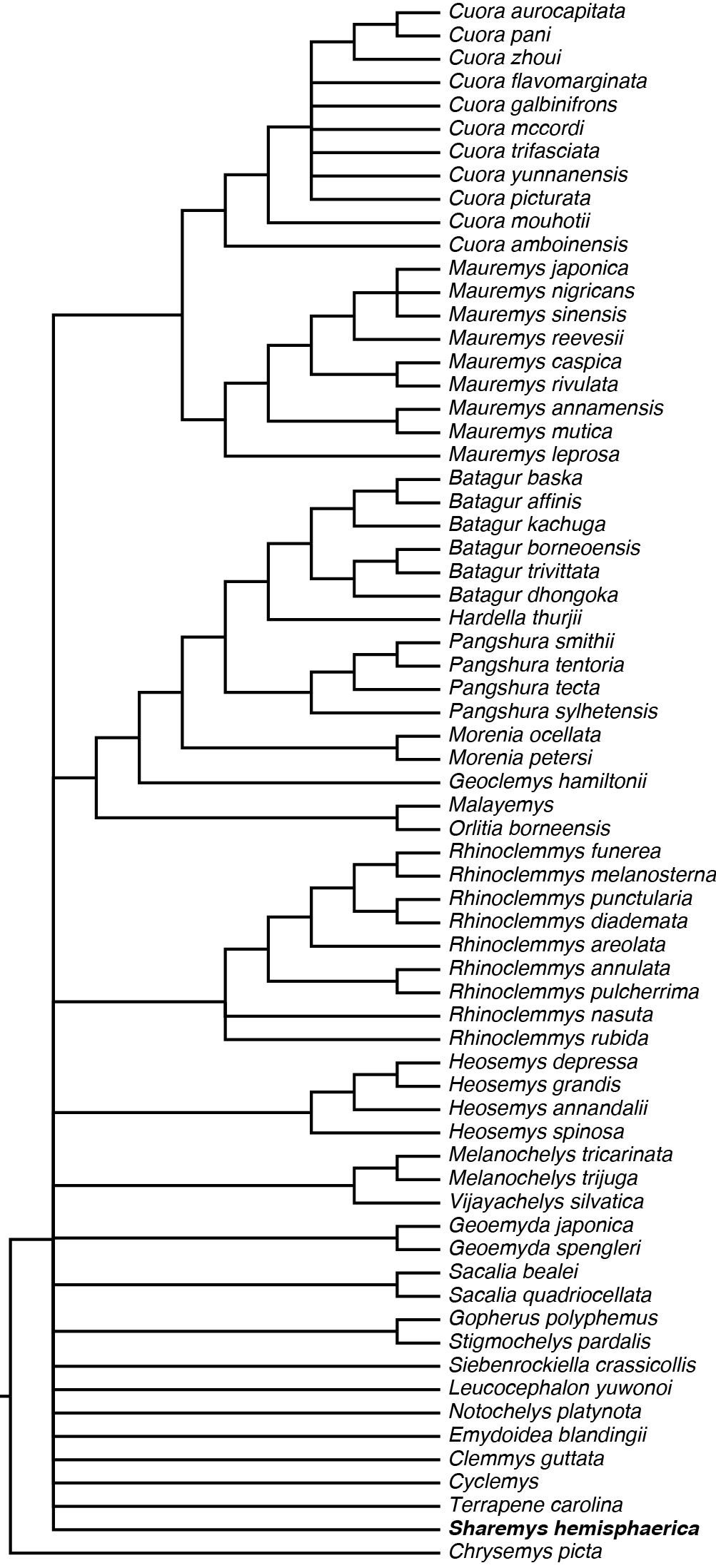


Figure S5.8: Strict consensus of total-evidence analysis with inclusion of the putative fossil testudinoid *Sharemys hemisphaerica*.


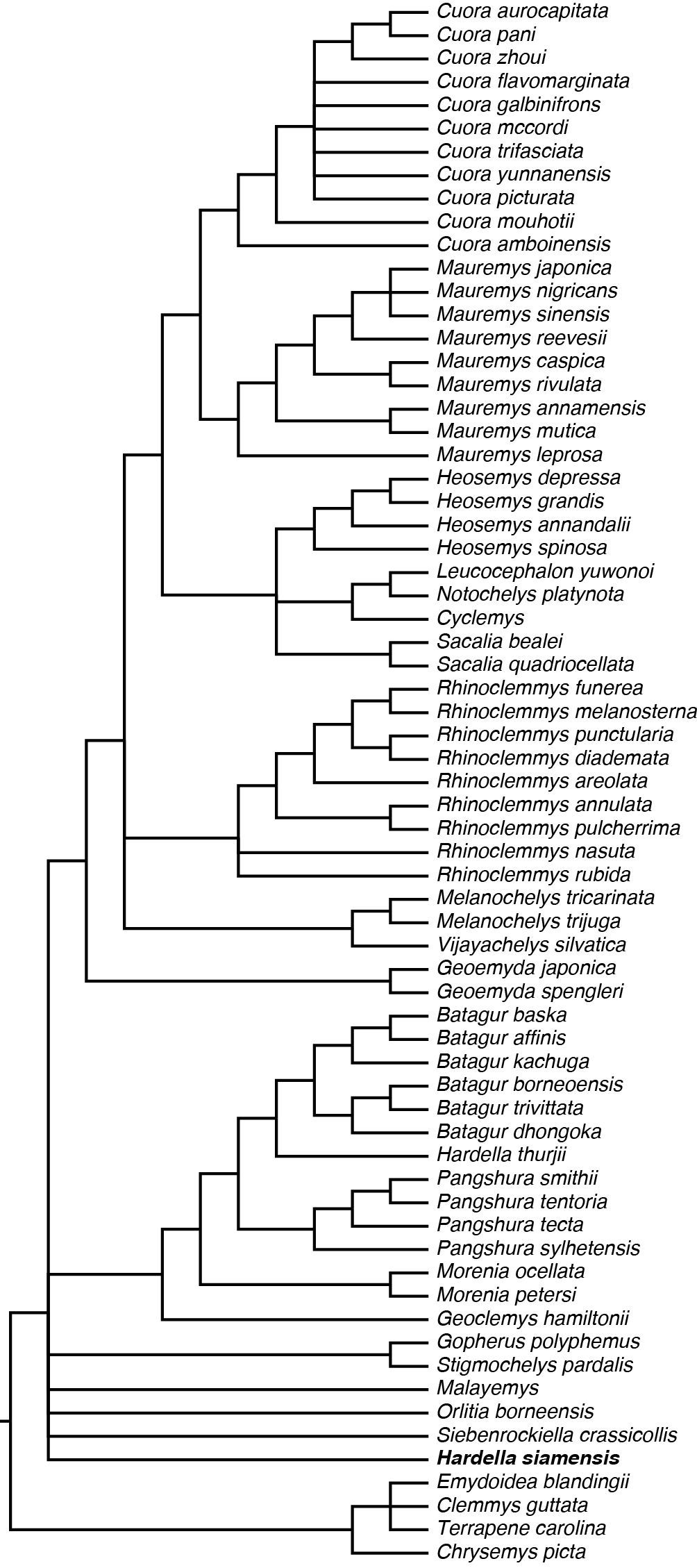


Figure S5.9: Strict consensus of total-evidence analysis with inclusion of the putative fossil geoemydid *Hardella siamensis*.


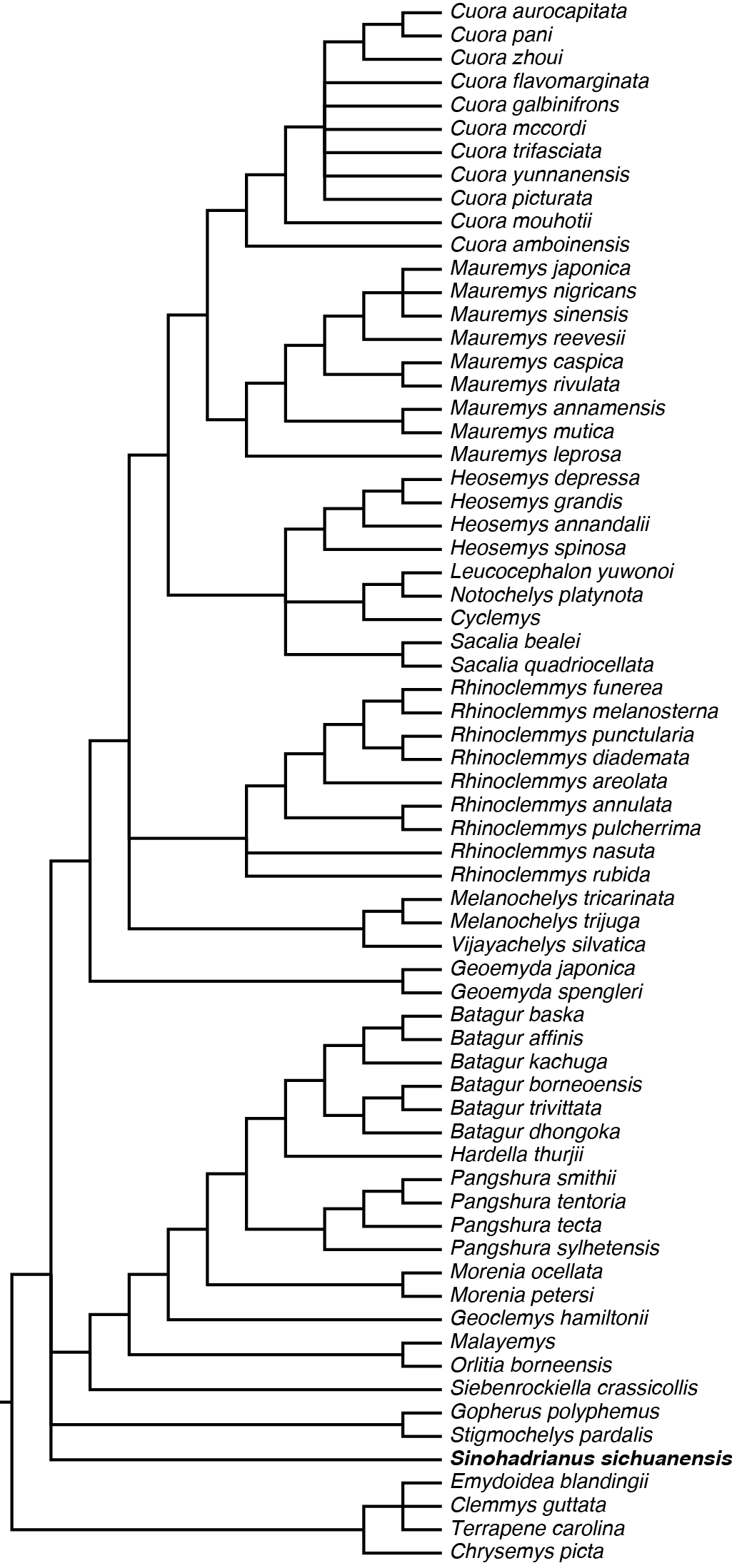


Figure S5.10: Strict consensus of total-evidence analysis with inclusion of the putative fossil testudinoid *Sinohadrianus sichuanensis*.

Figure S11: Strict consensus of total-evidence analysis with inclusion of the putative fossil geoemydid *Mauremys thanhinensis*.


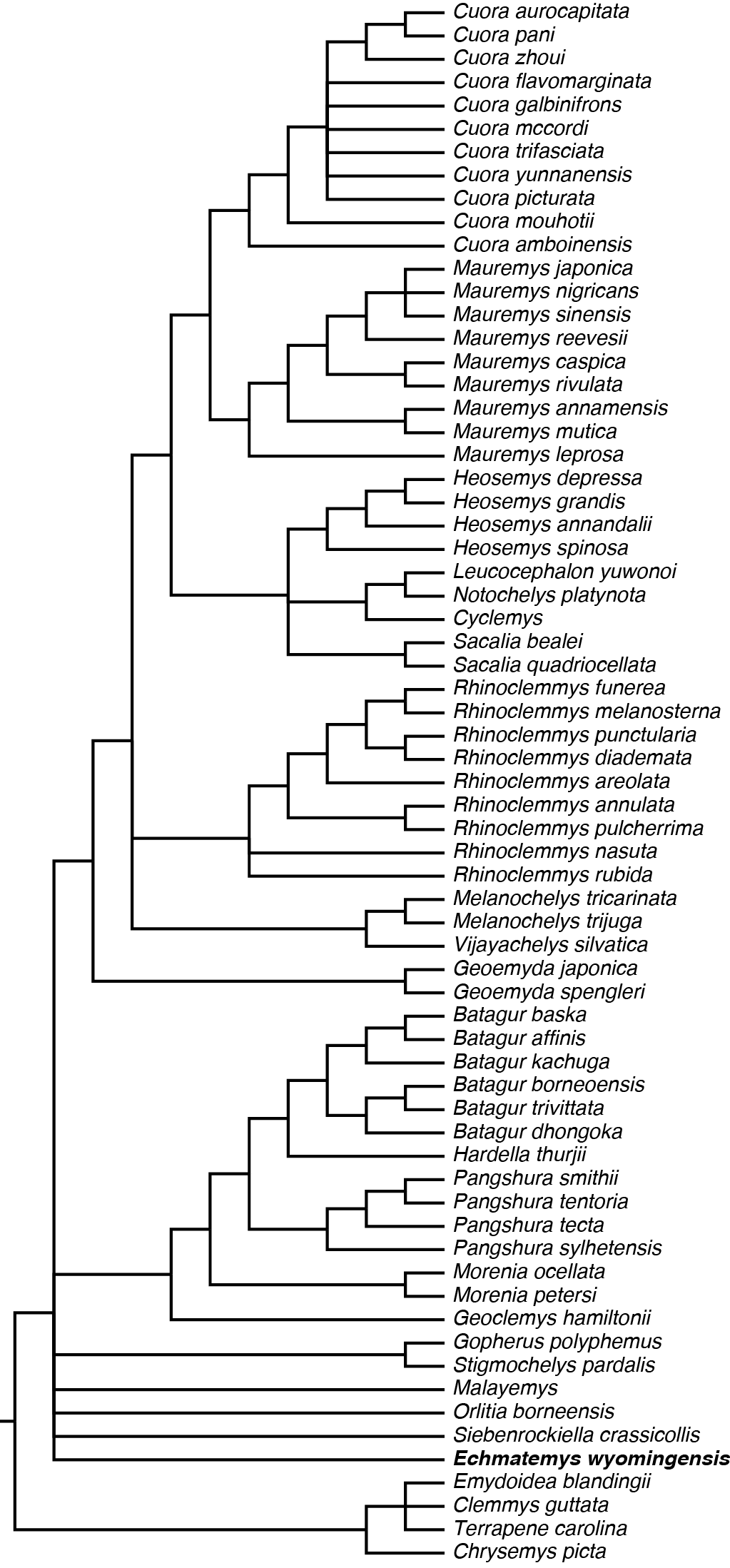


Figure S12: Strict consensus of total-evidence analysis with inclusion of the putative fossil geoemydid *Echmatemys wyomingensis.*
