# Supplementary figures and images for: A new testudinoid turtle from the middle to late Eocene of Vietnam
Source: PeerJ. 2019 Feb 18;7:e6280. doi: 10.7717/peerj.6280 (PMC6383559; doi:10.7717/peerj.6280)

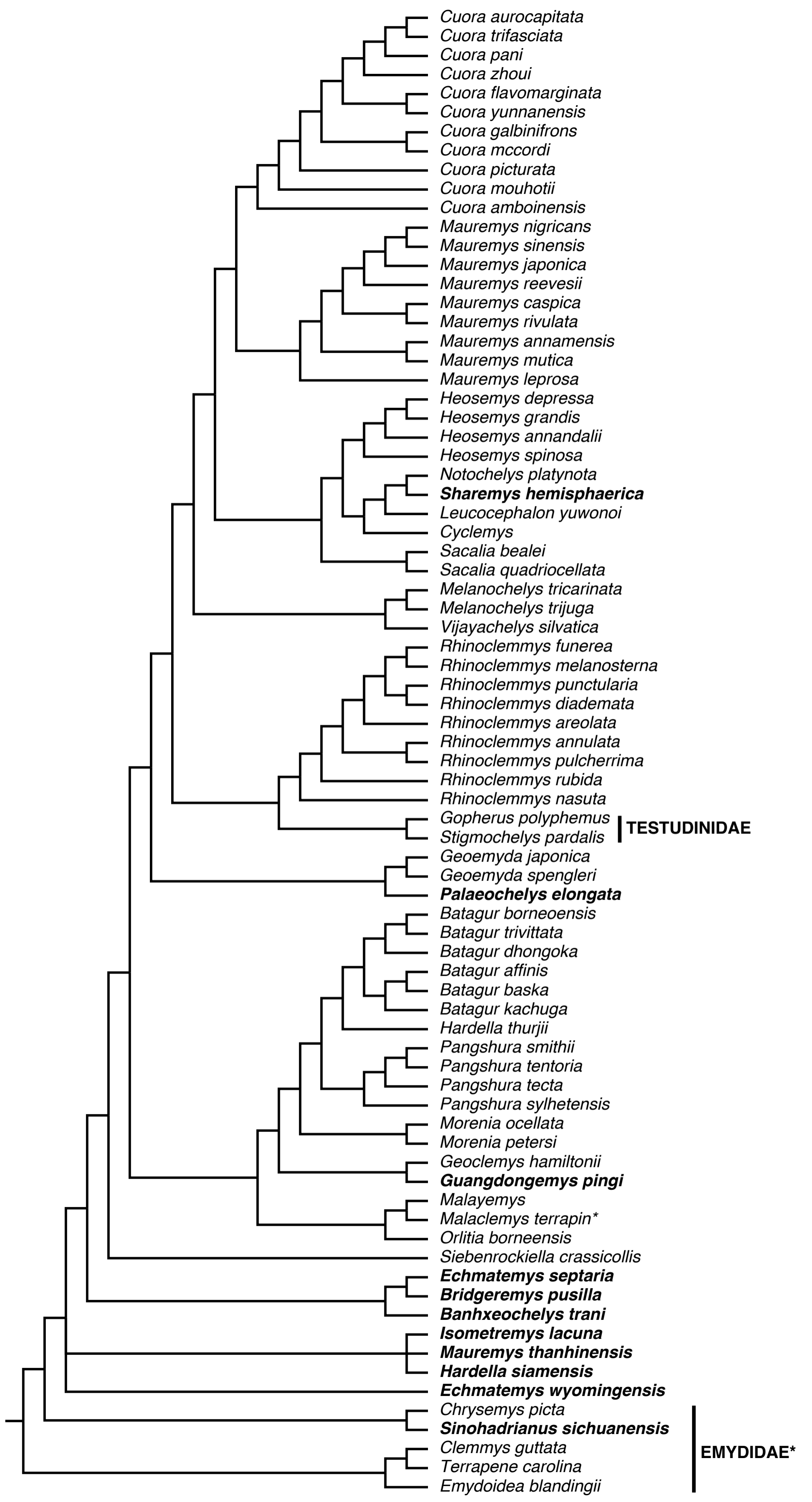

Supplement: Supplemental Information 7 — Fossils species are marked in bold lettres. Emydid species Malaclemys terrapin is marked with an asterisk to sinalize its position in the ingroup. [file peerj-07-6280-s007.pdf]
